# Impact of contact on adolescents’ mental health literacy and stigma: the SchoolSpace cluster randomised controlled trial

**DOI:** 10.1136/bmjopen-2015-009435

**Published:** 2016-02-19

**Authors:** Katharine Chisholm, Paul Patterson, Carole Torgerson, Erin Turner, David Jenkinson, Max Birchwood

**Affiliations:** 1 School of Psychology, University of Birmingham, Birmingham, UK; 2 Youth Programme, Birmingham and Solihull Mental Health NHS Foundation Trust, Research & Innovation, Birmingham, UK; 3 School of Education, University of Birmingham, Birmingham, UK; 4 Early Intervention Services, Birmingham and Solihull Mental Health NHS Foundation Trust, Newington Resource Centre, Birmingham, UK; 5 School of Health and Population Sciences, University of Birmingham, Birmingham, UK

**Keywords:** MENTAL HEALTH, PUBLIC HEALTH

## Abstract

**Objectives:**

To investigate whether intergroup contact in addition to education is more effective than education alone in reducing stigma of mental illness in adolescents.

**Design:**

A pragmatic cluster randomised controlled trial compared education alone with education plus contact. Blocking was used to randomly stratify classes within schools to condition. Random allocation was concealed, generated by a computer algorithm, and undertaken after pretest. Data was collected at pretest and 2-week follow-up. Analyses use an intention-to-treat basis.

**Setting:**

Secondary schools in Birmingham, UK.

**Participants:**

The parents and guardians of all students in year 8 (age 12–13 years) were approached to take part.

**Interventions:**

A 1-day educational programme in each school led by mental health professional staff. Students in the ‘contact’ condition received an interactive session with a young person with lived experience of mental illness.

**Outcomes:**

The primary outcome was students’ attitudinal stigma of mental illness. Secondary outcomes included knowledge-based stigma, mental health literacy, emotional well-being and resilience, and help-seeking attitudes.

**Results:**

Participants were recruited between 1 May 2011 and 30 April 2012. 769 participants completed the pretest and were randomised to condition. 657 (85%) provided follow-up data. At 2-week follow-up, attitudinal stigma improved in both conditions with no significant effect of condition (95% CI −0.40 to 0.22, p=0.5, d=0.01). Significant improvements were found in the education-alone condition compared with the contact and education condition for the secondary outcomes of knowledge-based stigma, mental health literacy, emotional well-being and resilience, and help-seeking attitudes.

**Conclusions:**

Contact was found to reduce the impact of the intervention for a number of outcomes. Caution is advised before employing intergroup contact with younger student age groups. The education intervention appeared to be successful in reducing stigma, promoting mental health knowledge, and increasing mental health literacy, as well as improving emotional well-being and resilience. A larger trial is needed to confirm these results.

**Trial registration number:**

ISRCTN07406026; Results.

**Strengths and limitations of this study**

- Although intergroup contact is a popular method to reduce the stigma of mental illness, this is the first study using a robust randomised controlled trial design to investigate intergroup contact combined with education compared with education alone.
- Much of the existing research concentrates on age groups ranging from mid to late adolescence, however, development of stigmatising attitudes and behaviours occurs in childhood and early adolescence, so it is vital that interventions for these age groups are investigated.
- Schools were chosen to represent the diversity of the UK school system in order to increase generalisability.
- Just two aspects of stigma were investigated; knowledge and attitude-based stigma. Other aspects of stigma, such as perceptions of dangerousness, otherness, or unpredictability were not investigated, and may interact differently with the impact of contact.
- Students reported that the intervention was well received and highly acceptable, however, acceptability of the intervention was assessed in just one school.

## Introduction

A majority of young people who develop mental health difficulties report experiencing stigma from their peers.^1^ The UK's ‘Time to Change’ programme is a large-scale national antistigma programme, which aims to reduce the stigma of mental illness by facilitating intergroup contact between the general public and individuals who experience mental disorders. Research from Time to Change describes the far-reaching consequences of stigma, with young people who experience mental disorders reporting that stigma had stopped them going to school (40%), socialising with friends (54%), or had led them to consider suicide (26%).^2^

Intergroup contact theory suggests that interaction between different groups reduces conflict, prejudice and discrimination.^3^ Contact interventions involve individuals with experience of living with a mental illness speaking about those experiences to members of the general population. Interventions may target stigma of a particular disorder (eg, depression), or may be more generic (‘mental illness’). Contact is often combined with education programmes, but can also act as a stand-alone intervention. Griffiths *et al*'s^4^ meta-analysis found that education interventions and contact interventions are effective in reducing stigma, but stated that there were very few randomised controlled trials (RCTs) which used contact. Corrigan and Penn^5^ suggest a combination of contact and education may offer the best opportunity for reducing stigmatising attitudes, and contact has become a successful component in antistigma campaigns.^6^ A recent meta-analysis comparing interventions which use contact alone, or contact plus education, to those which have used education alone, however, found that in adolescent populations, education alone may be a better strategy.^7^ The three studies which have *directly* compared contact and education with education alone, however, found contact following education significantly reduced stigma compared with education alone.^8–10^ Importantly, these studies also focused on mid-to-late adolescent age ranges. Targeting younger adolescent populations has a number of potential benefits. Stigmatising attitudes begin to form in childhood and early adolescence,^11–13^ meaning that interventions targeted at these age groups may have a more preventative role than those targeted at older individuals. Similarly, stigma, and a lack of knowledge or ‘mental health literacy’,^14^ has also been linked to a chronic delay in help-seeking,^15–18^ with only a minority of young people experiencing a diagnosable mental disorder accessing professional help.^16^ ^19^ As prevalence for the development of many mental disorders peaks in adolescence and early adulthood,^20^ targeting stigma earlier may help to reduce this delay.

Contact interventions aiming to improve stigma and literacy have not generally investigated mental health and well-being outcomes. There is emerging evidence, however, that school-based programmes which aim to reduce stigma and increase literacy may additionally improve participants’ mental health and resilience.^17^ Resilience can be considered as factors which may protect against the development of a mental illness, such as personal disposition, family cohesion and social support.^21^ Programmes which promote mental health and resilience tend to show greater impact than those which aim to reduce mental illness.^22^ Mental health literacy programmes which have a focus on increasing help-seeking and understanding of resilience skills such as self-esteem, may play into this.^17^ Contact may, additionally, help engagement with programmes, as adolescents report that they would value hearing personal experiences when being taught about mental health.^23^

This cluster RCT aimed, first, to test the hypothesis that contact, in addition to education, is more effective than education alone in reducing stigma, improving mental health literacy, and promoting well-being in young adolescents, and second, to assess the feasibility of conducting contact-based intervention research in an adolescent population, the ability of the facilitators to conduct the intervention with fidelity, and the acceptability of the contact element of the intervention to adolescent groups.

## Method

### Design

A pragmatic cluster RCT was undertaken in six secondary schools in Birmingham, UK. The full project protocol is described in Chisholm *et al.*^24^ The intervention was designed and reported in accordance with CONSORT guidelines.^25^

### Participants

Schools in Birmingham, UK, were approached based on specified criteria in order to represent the diversity of the UK school system and the socioeconomic and sociocultural strata of Birmingham (table 1). Once a school had consented to take part in the research, consent letters were sent to parents or guardians of all students in the participating year group. Schools were recruited, and the intervention implemented between April 2011 and April 2012.

**Table 1:** Criteria used to select schools

Criteria	Defined by
Type of school	Independent (fee paying), grammar (exam entry), comprehensive (open-access)
Socioeconomic profile of school	Percentage of pupils with free school meals
Intake profile of school	Ethnicity, gender and percentage of pupils with English as a second language
Geographic location of school	North, east, south and west Birmingham, UK

### Randomisation

Classes, rather than schools, were randomised in order to maintain power. Random allocation was concealed, generated by a computer algorithm, and undertaken after pretest. Each class within a school was given an identification number which was then emailed to an independent researcher at Birmingham and Solihull Mental Health NHS Foundation Trust who undertook the randomisation. Blocking was used to randomly stratify classes equally to condition within each school. Condition allocation was concealed from the statistician (DJ) in charge of devising the analysis. Condition allocation could not be masked from participants, teachers and intervention leads.

### Procedure

Two weeks prior to the intervention day, students with parental consent were invited to complete the self-report study measures during their class registration. Students indicated assent by checking a box and generated a code^26^ on their questionnaire, which was used to match an individual's responses over time and to the condition that the participant was randomised. Participants completed the same questionnaire two weeks postintervention, again during class registration. In two schools, participants also completed study measures at 6-month follow-up (see online supplementary tables S1–S4).

### The intervention

The authors (KC, PP and ET) developed the intervention using results from local surveys and focus groups, in collaboration with teachers and service-users. Additional resources evolved from the work of O'Reilly^27^ and the Staffordshire Changes Young People's mental health programme. Contact modules for the intervention were designed in collaboration with current and past users of mental health services. The young person with experience of mental illness, or ‘Contact Volunteer’, worked with the class throughout the morning, but did not reveal that they lived with a mental illness. Halfway through the day it was disclosed to the class that one of the people leading the intervention had experienced a mental illness. This was done so that the participants would be able to spend the morning getting to know the individuals without preconceptions based on the knowledge that they had a diagnosis. For the 20 min Contact Session, the Contact Volunteer then discussed what it is like to live with a mental illness, and answered questions from the class. The length of time for the formal contact presentation was decided on after discussion with the Contact Volunteers. The volunteer then continued to work with the class for the rest of the day and to discuss their experiences and answer questions in a less formal manner.

The majority of Contact Volunteers were recruited via the Early Intervention in Psychosis Service. Other individuals were recruited via the Youthspace Programme (http://www.youthspace.me) and service-user research groups from the Mental Health Research Network. Individuals had a range of different experiences and diagnoses including psychosis, depression, anxiety disorders and borderline personality disorder. The most prevalent experience was of psychosis.

Interventions followed the same lesson plans with the exception of a 20 min ‘contact module’ in the contact condition, and a 20 min ‘history of mental health module’ in the education condition (table 2).

**Table 2:** Intervention lesson plans

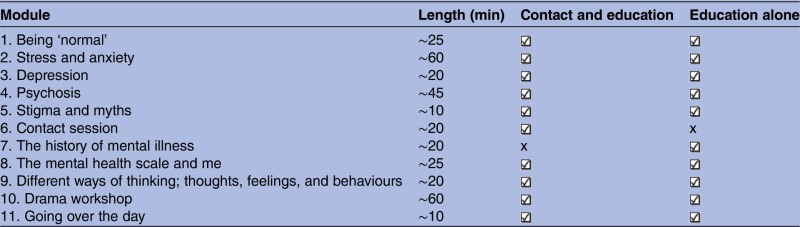

∼, approximately.

Interventions were led by staff from Birmingham and Solihull Mental Health Foundation NHS Trust along with other trained volunteers, some of whom had experience of mental illness. The intervention days were coordinated by KC, PP and ET, and overseen by MB.

### Outcomes

#### Primary outcome

##### Stigma of mental illness

The Reported and Intended Behaviour Scale (RIBS)^28^ takes approximately 1–2 min to complete, and generates a score based on willingness to have contact with individuals who are experiencing mental illness (‘In the future I would be willing to live with someone with a mental health problem’). Scores on the RIBS range from 4 to 20, with higher scores indicating more positive attitudes. Within adult groups, the RIBS has a test–retest reliability of 0.75, and a Cronbach's α of 0.85.

#### Secondary outcomes

##### Knowledge of mental illness

Knowledge-based stigma was assessed using the Mental Health Knowledge Schedule (MAKS).^29^ The MAKS assesses six domains of stigma-related knowledge: help-seeking, recognition, support, employment, treatment, and recovery, and takes 1–2 min to complete. Scores range from 12 to 60. Higher scores indicate a higher level of knowledge. The MAKS has a test–retest reliability of 0.71, and has been extensively reviewed by experts. The MAKS Cronbach's α is moderate at 0.65. This is largely due to the fact that the MAKS is not intended to function as a scale; individuals may have different levels of knowledge based on different domains. Two vignettes were used to assess mental health literacy, specifically, identification of mental illnesses, developed by Jorm *et al.*^14^ Participants were asked ‘In the above story do you think John/Peter has…’ and chose from answers ‘depression’, ‘anxiety’, ‘psychosis or schizophrenia’, ‘drug addiction’, or ‘no mental health problems’. A score of 1 was given if the correct mental disorder was identified.

##### Emotional well-being

The Strengths and Difficulties Questionnaire (SDQ)^30^ was used to assess mental health. The SDQ assesses health and vulnerabilities on five subscales (conduct problems, hyperactivity-inattention, emotional symptoms, peer problems, and prosocial behaviour) and produces a total difficulties score. The SDQ has been validated for use with adolescents aged 11–16 years with a Cronbach's α of 0.82 for the total difficulties scale. Scores range from 0 to 40, and higher scores indicate lower levels of mental health.

##### Resilience

Resilience was measured using a 15-item^31^ version of the Resilience Scale,^32^ which assesses the personal competence component of resilience (‘My belief in myself gets me through hard times’). The scale has reported Cronbach's αs of between 0.72 and 0.94, and has been used previously with adolescent populations.^31^ ^33^ Scores range from 15 to 105. Higher scores indicate a higher level of resilience.

##### Help-seeking

Attitudes to help-seeking were assessed by responses on a seven-point scale to the question ‘In the next 12 months if you were to experience a mental illness, how likely are you to seek help?’. Higher scores indicate a greater willingness to help-seek.

##### Acceptability

Acceptability of the intervention, including method of delivery and content, was assessed in one school (school 2) by author KC. Two weeks postintervention, students who had attended the intervention day took part in two short group interviews^34^ of 5–6 participants. Interviews were recorded and transcribed verbatim. The interview schedule can be seen in box 1.

Box 1 **Semistructured interview schedule**

Focal points for group interview

1. Was there anything on the course that you thought was particularly good or useful?
2. Was there anything that you thought should have been on the course that was not?
3. Are there any ways in which the course could be made better?

##### Fidelity of implementation

A day's training was provided for all individuals facilitating the intervention. One class per condition, per school, was assessed for fidelity between conditions and schools by KC with a predeveloped checklist which measured pace and timing of the intervention, engagement of students, and group work.

### Analysis

An intraclass correlation coefficient (ICC) of 0.037 (Aberdeen University: Health Services Research Unit) was assumed, and a cluster size of approximately 30 students per class, suggesting that 738 participants would be needed to detect a Cohen's d effect size of 0.3. The rationale behind aiming to detect an effect size of 0.3 was that previous research in school-based studies has often found relatively small effect sizes,^7^ which, nonetheless, may be meaningful in population-based samples.

To investigate the primary research question, data was analysed with generalised equation estimates (GEE) in SPSS, V.20. In accordance with CONSORT guidelines, unadjusted analysis was employed as the primary analysis. In order to account for the clustered nature of the RCT, school and condition were included as covariates, as well as baseline measure scores. The GEE was also used to accommodate the fact that data on which class each participant was in was not collected, meaning that the analysis was unable to account for this aspect of the clustering. Outcomes were transformed if skewed. Where data was ordinal an ordinal logistic GEE was used. An adjusted analysis was also employed, with gender, ethnicity, previous contact, and whether the participant reported having been diagnosed with a mental health disorder added as additional factors. Intention-to-treat analysis was used.

To assess any change in participants’ scores, preintervention to postintervention Student t tests, or marginal homogeneity tests (where data was ordinal), were employed. Cronbach's αs were computed for all measures. An analysis of percentage of items left unanswered for each item from each questionnaire assessed acceptability of the measures. The ICC was calculated on the baseline RIBS scores. The method used was the one based on the analysis of variance, with the CI being calculated using Searle's method (adjusted for unequally sized clusters), as given in ref. 35.

## Results

Participants were recruited between 1 May 2011 and 30 April 2012. Six schools and 31 classes took part in the intervention. Demographic characteristics of schools can be seen in table 3.

**Table 3:** Demographic characteristics* of schools

	School type	Students aged 5–15 years	Classes per year group	Students with English second language (%)	Students with free school meals (%)	Ethnicity			
						South Asian (%)	White (%)	Black (%)	Other (%)
1	Mixed comprehensive school	1288	7	9	22	9	79	4	8
2	Girls only grammar school	668	4	23	6	45	35	10	10
3	Mixed comprehensive school	798	6	18	54	8	65	14	13
4	Boys only comprehensive school	611	5	26	30	35	47	6	12
5	Girls only comprehensive school	635	4	78	48	71	3	19	7
6	Boys only grammar school	622	5	23	4	28	59	4	9

*Data available from Birmingham City Council, accessed 2009.

**Figure 1:**
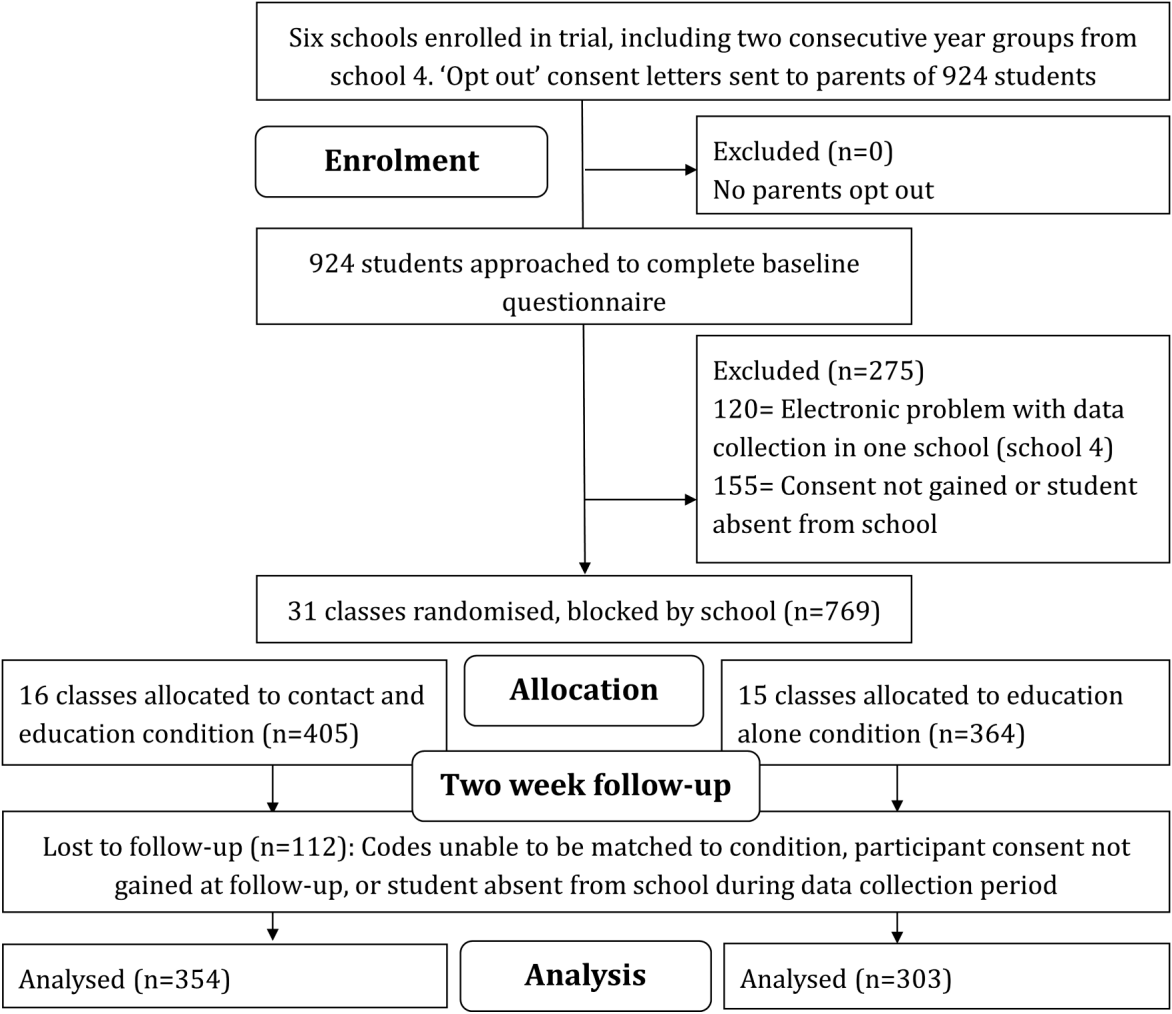
Participant enrolment, allocation, follow-up and analysis for main trial.

In total, 769 participants provided data at baseline. Of these, 112 were absent for the intervention day or were lost to follow-up; 657 participants aged 11–13 years (mean 12.21, SD 0.58) took part in the trial. Baseline characteristics of participants can be seen in table 4. Baseline and 2-week means, SDs, medians and significance of improvement between baseline and 2 weeks can be seen in table 5. A summary of the effect between conditions at 2 weeks can be seen in table 6, for the primary unadjusted analysis and the adjusted analysis which used gender, ethnicity, previous contact, and whether the participant reported having been diagnosed with a mental illness, added as additional factors. The CONSORT diagram is presented in figure 1.

**Table 4:** Baseline characteristics between conditions

		Gender		Ethnicity						Current mental health diagnosis		Previous contact	
Condition	Total *N*	Male	Missing	White	Asian	Black	Mixed ethnicity	Other ethnicity	Missing	Yes	Missing	Yes	Missing
Contact and education													
N	354	171	0	149	141	29	23	9	3	10	7	92	8
%	100	48.30	0	42.10	39.80	8.20	6.50	2.50	0.80	2.80	2	26	2.30
Education only													
N	303	144	0	119	127	19	27	8	3	4	4	77	5
%	100	47.50	0	39.30	41.90	6.30	8.90	2.60	1	1.30	1.30	25.40	1.70

**Table 5:** Significance of change; baseline—2 weeks

	Pre		2 weeks		t/z Value	95% CI	p Value
	Mean (SD)	Median	Mean (SD)	Median			
RIBS							
C&E	13.28 (3.71)	13	13.81 (3.96)	14	−3.84	−0.99 to −0.32	<0.001
E	13.10 (4.29)	14	13.85 (3.83)	14	−3.62	−1.21 to −0.36	<0.001
MAKS							
C&E	39.92 (3.86)	40	42.98 (5.77)	43	−8.91	−3.90 to −2.49	<0.001
E	40.25 (4.04)	40	43.28 (5.83)	44	−9.50	−4.52 to −2.96	<0.001
Vignettes							
C&E	1.19 (0.74)	1	1.23 (0.77)	1	−1.03	–	0.3
E	1.18 (0.72)	1	1.32 (0.73)	1	−2.49	–	0.01
SDQ							
C&E	9.69 (5.63)	9	9.15 (5.90)	8	2.31	0.02 to 0.19	0.02
E	9.72 (5.57)	9	8.87 (5.87)	8	4.81	0.12 to 0.29	<0.001
Help-seeking							
C&E	5.41 (1.71)	6	5.51 (1.67)	6	−0.92	–	0.4
E	5.35 (1.71)	6	5.48 (1.62)	6	−1.24	–	0.2
Resilience							
C&E	83.88 (13.38)	86	82.50 (15.75)	86	0.86	−0.11 to 0.28	0.4
E	82.80 (13.79)	85	83.34 (15.47)	85	2.87	0.07 to 0.39	0.005

*Significance of change for the Reported and Intended Behaviour Scale (RIBS), Mental Health Knowledge Schedule (MAKS), mental health literacy (vignettes), the Strengths and Difficulties Questionnaire (SDQ), help-seeking, and resilience. C&E, contact and education; E, education alone.

**Table 6:** Effect of condition at 2 weeks, unadjusted and adjusted GEEs

	Contact and education		Education alone		Model	Treatment effect for contact plus education	95% CI	p Value
Measure	Mean (SD)	Median	Mean (SD)	Median				
RIBS	13.81 (3.96)	14	13.85 (3.83)	14	Unadjusted	−0.09	−0.40 to 0.22	0.5
					Adjusted	−0.07	−0.41 to 0.28	0.7
MAKS	42.98 (5.77)	43	43.28 (5.83)	44	Unadjusted	−0.65	−1.13 to −0.17	0.008
					Adjusted	−0.72	−1.28 to −0.16	0.01
Vignettes	1.23 (0.77)	1	1.32 (0.73)	1	Unadjusted	−0.30	−0.44 to −0.16	<0.001
					Adjusted	−0.35	−0.47 to −0.23	<0.001
SDQ	9.15 (5.90)	8	8.87 (5.87)	8	Unadjusted	0.10	−0.01 to 0.18	0.02
					Adjusted	0.11	0.02 to 0.19	0.01
Help-seeking	5.51 (1.67)	6	5.48 (1.62)	6	Unadjusted	−0.26	−0.52 to −0.00	0.05
					Adjusted	−0.20	−0.41 to 0.01	0.07
Resilience	82.50 (15.75)	86	83.34 (15.47)	85	Unadjusted	0.19	−0.15 to 0.52	0.3
					Adjusted	0.16	−0.16 to 0.48	0.3

*Effect of condition at 2 weeks for the Reported and Intended Behaviour Scale (RIBS), Mental Health Knowledge Schedule (MAKS), mental health literacy (vignettes), the Strengths and Difficulties Questionnaire (SDQ), help-seeking, and resilience.

The unadjusted GEE, 0.09, 95% CI (−0.40 to 0.22), p=0.5, Cohen's d=0.01, found no significant effect of condition on participants’ attitudinal-based stigma at 2-week follow-up. Contrary to the hypothesis, participants’ knowledge-based stigma in the education-alone condition improved significantly more than participants in the contact and education condition, −0.65, 95% CI (−1.13 to −0.17), p=0.008, d=0.05. Similarly, an ordinal logistic GEE found that participants in the education-alone condition displayed greater improvement in mental health literacy 2 weeks postintervention compared with participants in the contact and education condition, −0.30, 95% CI (−0.44 to −0.16), p<0.001, d=0.12.

A square root transformation was employed for emotional well-being, baseline and follow-up data. The unadjusted GEE revealed that postintervention participants in the education-alone condition had greater improvements in levels of emotional well-being compared with participants in the contact and education condition, 0.10, 95% CI (0.01 to 0.18), p=0.02, d=0.05. Similarly, an ordinal logistic GEE found that participants in the education-alone condition displayed greater improvements in their willingness to help-seek compared with participants in the contact and education condition, −0.26, 95% CI (−0.52 to −0.00), p=0.05, d=0.02. Finally, resilience data was reverse-coded, and a square root transformation was used on baseline and follow-up data. The unadjusted GEE found no significant difference in improvement between conditions at follow-up, 0.19, 95% CI (−0.15 to 0.52), p=0.3, d=0.05.

Student t tests, and marginal homogeneity tests, were employed to assess significance of change in participants’ scores preintervention to postintervention. Participants’ attitudinal-based stigma improved from baseline to follow-up (see table 5 for means). These improvements were found to be significant for both the contact and education condition, t (255)=−3.84, 95% CI (−0.99 to −0.32), p<0.001, Pearson's r=0.23, and the education-alone condition, t (193)=−3.62, 95% CI (−1.21 to −0.36), p<0.001, r=0.25. Knowledge-based stigma also improved significantly for participants in the contact and education condition, t (195)=−8.91, 95% CI (−3.90 to −2.49), p<0.001, r=0.54, and the education-alone condition, t (169)=−9.50, 95% CI (−4.52 to −2.96), p<0.001, r=0.59. In the contact and education condition, improvement in mental health literacy scores was not significant, z=−1.03, p=0.3, r=0.05. Conversely, participants in the education-alone condition demonstrated a significant improvement in mental health literacy at follow-up, z=−2.49, p=0.01, r=0.13.

Participants’ emotional well-being scores improved significantly for the contact and education condition, t (194)=2.31, 95% CI (0.02 to 0.19), p=0.02, r=0.16, as well the education-alone condition, t (165)=4.81, 95% CI (0.12 to 0.29), p<0.001, r=0.35. Participants’ resilience scores improved significantly in the education-alone condition, t (157)=2.87, 95% CI (0.07 to 0.39), p=0.005, r=0.22. In the contact and education condition, resilience scores decreased, but not significantly; t (152)=0.86, 95% CI (−0.11 to 0.28), p=0.4, r=0.07. For help-seeking, no significant change preintervention to postintervention was found for the contact and education condition, z=−0.92, p=0.4, r=0.05, or the education-alone condition, z=−1.24, p=0.2, r=0.07.

Participants reported finding the intervention highly acceptable. In particular, the use of contact, interactive methods of delivery, and expert and friendly presenters were praised. Areas suggested for improvement were ensuring language and explanations were clear and age appropriate, making sure time was allowed for class discussion, more information on help-seeking avenues, and more information on violence in mental illness. Quotes are presented in table 7, and highlight participant views.

**Table 7:** Quotes highlighting participants’ feedback on the intervention

Positive elements	
Intergroup contact	‘The talk with Camilla was the most helpful thing because it was like, you probably like, you probably weren't ever going to talk to a mental um person like someone who's actually been there, done that kind of thing. So you probably won't get the chance and like if like it was good cos then you knew what people go through’. ‘A stereotype of a crazy person, um someone with a mental illness is someone who's crazy, speaks nonsense, but she looked really normal. So that just goes to show that people with mental illnesses are normal, but in their own way’.
Presenters	‘They were very like straight to the point and they didn't over exaggerate it either’. ‘They were chatty, they didn't just read off the board, they spoke to you like not in a boring way just didn't waffle’. ‘They didn't scare you but they made you understand’.
Interactive elements	‘I liked the videos because they were effective and they actually showed you what people can do’. ‘I liked the true and false one where you had to see where, cos you were still learning then, but like without having to just sit there. it gets you more interactive so you feel like you're actually taking part in that’. ‘I liked the drama as well because it was like um it was almost like, cos we were doing stress and I think Mika, cos I was Mika in one of them, Mika was stressed, so you kind of like, you learnt what stress is actually like’.
Areas for improvement	
Language and explanations	‘In the end, they kind of kept saying what is normal and I couldn't really put my finger on it—is everyone normal? Is no-one normal? And it really like made my brain fuzzy, it's really hard to think straight. I did find it useful, it was just really difficult’. ‘I didn't find it, the drama bit boring because it was really funny watching it, like everyone in the class watching, but the bit afterwards because it, it used words that I didn't understand like ‘bodily language’’.
Time for discussion and questions	‘I found that we just got loaded on with information more than discussed it’.
Help-seeking	‘More on what you could do if you like did have mental illness because you could see a doctor or you could er go on this website to get help but they didn't really tell us anything else that we could do’.
Violence	‘What triggers them to be dangerous?’

For the primary outcome, an ICC of 0.10, 95% CI (0.04 to 0.26) was found. Cronbach's αs, in the present sample, were 0.86 for the RIBS, 0.24 for the MAKS, 0.72 for the SDQ, and 0.89 for the Resilience scale. The items missing analysis revealed a high level of acceptability for the measures used, with no items standing out as being left unanswered by the majority of participants. Percentage of items left unanswered by participants for the RIBS ranged from 0% (In the future, I would be willing to work with someone with a mental health problem) to 0.7% (In the future, I would be willing to continue a relationship with a friend who developed a mental health problem), for the MAKS from 0.8% (most people with mental health problems want to have paid employment) to 5.1% (drug addiction is a type of mental illness), for the SDQ from 0.4% (I usually share with others) to 6.9% (I get on better with adults than with people my own age), and for the Resilience Scale from 0.5% (when I make plans I follow through with them) to 5.7% (I usually take things in my stride).

Facilitators demonstrated a high level of fidelity to the intervention, measured by a predeveloped checklist. All presentation slides were covered, and presenters moved at approximately equal speed through intervention modules (table 8). The majority of students in each school appeared to be engaged in the intervention, participating in group activities and joining in with group discussions.

**Table 8:** Fidelity of implementation observation checklist

Checklist item	Condition	Session observed	Outcome
Timing from first slide to class exercise	Contact	First module	10–12 min
	Education	Second module	10–26 min
Time allocated to first exercise	Contact	First module	8–13 min
	Education	Second module	5–10 min
Are any slides skipped?	Contact	First module	No, in all observed classes.
	Education	Second module	No, in all observed classes.
Are pupils asked if they have questions?	Contact	First module	Yes, in all observed classes.
	Education	Second module	Yes, in all observed classes.
Are the majority of students engaging in class?	Contact	First module	Yes, in all observed classes.
	Education	Second module	Yes, in all observed classes.
Size of group for first exercise	Contact	First module	2–7
	Education	Second module	3–5
Does a facilitator visit each group during exercise?	Contact	First module	Yes, in all observed classes.
	Education	Second module	Yes, in all observed classes.
Do all groups manage to finish exercise in allocated time?	Contact	First module	Yes in 4 observed classes, no in 2 observed classes.
	Education	Second module	Yes, in all observed classes.

## Discussion

The current study found that for an educational intervention within a young adolescent population, contrary to study hypothesis, intergroup contact did not add value to education alone in improving attitudinal stigma of mental illness. Similar results to these were found for the secondary outcome of resilience, with intergroup contact adding no value to education alone. For secondary outcome measures of knowledge, emotional well-being and help-seeking participants’ scores in the education-alone condition improved significantly more than those in the contact and education condition.

The results are in line with the findings of a meta-analysis from Corrigan *et al*^7^ which compared education-alone interventions to contact interventions, and suggested that within adolescent populations, education interventions held more promise for the reduction of stigma. On the other hand, the findings conflict with the only three previous studies which investigated education and contact compared with education alone in adolescent populations.^8–10^ There are several possible reasons for the absence of gains from contact in this trial. The majority of research into the relationship between contact and stigma has been conducted within adult populations. Owing to the rapid nature of brain changes throughout adolescence^36^ ^37^ there may be a large discrepancy in level of maturation between adolescents who differ in age even by a year or two. Although only a few years’ age gap separates the young adolescents who took part in the present study from the slightly older adolescents of Meise *et al*,^8^ Chan *et al*^10^ and Husek,^9^ this developmental difference may have had an impact on participants’ response to contact. Pinto-Foltz *et al* 38 suggest that adolescents may conceptualise the term ‘mental illness’ in a different way to older populations. For example, young adolescents may lack an internal reference system for mental illness, or have a framework of mental illness which is somewhat undifferentiated. If this is the case, then contact may serve more to confuse than to clarify, as mental illness in ‘reality’ often does not conform neatly into diagnostic categories, and comorbidity is common.^39^ Alternatively, adolescents may have an internal framework for mental illness, but it may be a negative or fearful framework. Adolescents’ conception of mental health may be influenced by media representations of mental illness^40^ leading to a framework which encapsulates many negative extremes of mental illness. If the contact used in the intervention was successful in normalising mental illness then fear of developing an illness may have increased leading to cognitive avoidance strategies^41^ in participants as a defence mechanism against anxiety. Participants may have distanced themselves from the topic of mental illness, increasing their desire for social distance, and leading to a decreased engagement in the educational elements of the intervention, and a diminished impact on outcome variables.

One further hypothesis is that the contact module with its element of surprise had an amplified impact on students, leading to this section of the intervention being recalled over and above other modules. The contact module occurred midway through the day, and may have been particularly attention-grabbing, effectively wiping much of the educational elements of the intervention. Increased engagement in the contact module may have left participants with less attentional capacity to process other information presented, leading to decreased levels of improvement on the research measures when compared with the education-alone condition. This account is in line with themes discussed in the focus groups investigating the acceptability of the intervention, in which participants reported engaging with and valuing the contact. It is possible that the introduction of the contact was too sudden, and that contact may have had a more positive impact if introduced in a different manner, for example, after more time to consolidate the educational aspect of the intervention. If correct, this would suggest that it was not the contact per se which reduced the impact of the intervention, but the timing and manner in which the contact was introduced. Rusch *et al* 42 outline a number of factors which are advantageous if contact is to be successful including equal status and cooperative interaction between group members as well as institutional support. The current intervention had support from the senior management within the schools, and cooperative interaction was reached by the inclusion of group activities and discussions in which both students and ‘contact volunteers’ took part. Rusch *et al*'s criteria of ‘equal status’ was, however, not entirely possible, as the school environment naturally lends itself to a division of status between teacher and student.^42^ Rusch *et al* also discuss the need for members of the stigmatised group to disconfirm stereotypes only mildly, and suggest that individuals who disconfirm a stereotype too strongly may not have the desired effect of reducing stigma.^42^ Instead, participants may decide that the individual represents an ‘exception to the rule’. Some of the young people who shared their experiences with the students were partially recovered. This may have led participants to define them differently on a conceptual level to ‘mentally ill’ and reduced the overall impact of the contact.

There are a number of implications regarding the use of intergroup contact with young adolescent populations which are important for mental health policy and antistigma campaigns. The students participating in the current research had just a single morning session of mental health education directly prior to the contact element of the intervention, with no time in between to process the information they had received. If young adolescents do lack an internal reference system for mental illness it may be that they require more extensive mental health education prior to experiencing contact compared with adults.^10^ Although contact in the present intervention followed an educational component, it may be that due to the participants’ relatively young developmental stage, the quantity of education given prior to contact (approximately 3 h) was insufficient. Similarly, if the engaging experience of contact reduced attentional capacity for other intervention modules then contact may still prove to be an effective technique for reducing stigma in young people if additional time is given for participants to process the information they have received before the introduction of intergroup contact. Additionally, it is felt that adolescents may also need more time and discussion *after* the presentation of contact to consolidate and process the information they have received.^43^ To investigate this possibility, future research could occur over a number of sessions over several days, allowing for the consolidation of educational elements of the intervention before introducing contact elements. The current research suggests, however, that it would be premature to implement large-scale dissemination of contact as a means to reduce stigma in adolescent populations.

There is little previous research which examines the use of contact as a means to address well-being in adolescents. Where research has examined this question, it has usually been in relation to attitudes to help-seeking, with some authors reporting that the use of contact improved attitudes,^44^ and others, that no significant improvements were observed.^45^ An interesting outcome of the present research is that mental health improved despite the fact that much of the intervention dealt with topics unrelated explicitly to the promotion of mental health. Previous interventions aiming to improve mental health have had some success^22^ although others have reported flat results.^46^ Mental health literacy topics have a direct relevance to the promotion of mental health, through the raising of awareness of mental health subjects, resilience or coping mechanisms,^17^ and may prove to be a successful technique for increasing well-being in adolescents.

Previous research has been criticised for only representing specific school types (eg, fee-paying, single-gender schools).^47^ For the SchoolSpace Trial, intervention schools were chosen to represent the diversity of the UK school system in order to increase generalisability. Schools, therefore, may not have represented a homogeneous group, despite being analysed in this way. It is also important to note that the acceptability of the intervention was assessed in just one school, and that these results may, therefore, not generalise to other schools which took part in the study. To maintain power, classes were randomised within schools to each condition, rather than entire schools, which may have allowed a degree of cross-contamination between conditions, and magnified intraclass correlations. This means that effect sizes between conditions may have been diluted. The analysis design accounted for clustering by including school and condition (contact and education or education alone) as covariates. Data on which class each participant was in was not collected, meaning that the analysis was unable to account for this aspect of the clustering. In addition, the sample size achieved was small to moderate, which will have impacted the power of the study. Two of the studies measures, the RIBS and the MAKS, were not validated for use with adolescent populations. The Cronbach's α for the RIBS in the present sample was high, and an items missing analysis found that the measure was highly acceptable to participants. The Cronbach's α was low for the MAKS. Lower Cronbach's αs have also been found with adult samples.^29^ The authors of the MAKS suggest this is because individuals have different levels of knowledge based on the different domains that the MAKS covers. These differences are likely to be even more pronounced in adolescent samples, resulting in a low Cronbach's α. The items missing analysis of the MAKS found that the measure was acceptable to participants, with very few participants skipping items on the measure. It is important to acknowledge that the research investigated two aspects of stigma, intended behaviour towards individuals diagnosed with a mental illness, and stigma-based knowledge. Other aspects of stigma, such as perceptions of dangerousness, otherness, or unpredictability were not investigated, and may interact differently with the impact of contact. Fidelity of implementation of the intervention was assessed for each condition within each school; facilitators demonstrated a high level of fidelity to the intervention implementation, and similar levels of engagement were observed across conditions, representing a strength of the project.

The present research appears to demonstrate that short educational interventions provided in schools can be successful in reducing the stigma of mental illness, as well as improving mental health literacy and outcomes of well-being. Contrary to study hypothesis, intergroup contact was not seen to add value. This is important for those involved in developing mental health and educational policy aiming to reduce stigma, and increasing mental health literacy and well-being in adolescent populations, although further research into this area is certainly warranted.
